# Supplementary material for: Synergistic antibacterial effect of silver and ebselen against multidrug‐resistant Gram‐negative bacterial infections
Source: EMBO Mol Med. 2017 Jun 12;9(8):1165–78. doi: 10.15252/emmm.201707661 (PMC5538294; doi:10.15252/emmm.201707661)
Supplement: Supplementary file 1 — Appendix [file EMMM-9-1165-s001.pdf]

Synergistic antibacterial effect of silver and ebselen against multidrug-resistant Gram negative bacterial infections

Lili Zou, Jun Lu, Jun Wang, Xiaoyuan Ren, Lanlan Zhang, Yu Gao, Martin E. Rottenberg, Arne Holmgren

Appendix

Appendix Tables S1 – S4

Appendix Table S1. Clinical isolated multidrug-resistant Gram-negative strains used in this work

Strain	Description	Source
KP-1	*Klebsiella pneumonia* (*K. pneumonia*) *subsp. pneumonia* 13#	This work
KP-2	*K. pneumonia subsp. pneumonia* 0322#	This work
AB-1	*Acinetobacter baumannii* (*A. baumannii*) H#	This work
AB-2	*A. baumannii* 0361#	This work
PA-1	*Pseudomonas aeruginose* (*P. aeruginose*) 1298#	This work
PA-2	*P. aeruginose* 0009#	This work
ECL-1	*Enterobacter cloacae* (*E. cloacae*) 0431#	This work
ECL-2	*E. cloacae* 2301#	This work
ECO-1	*Escherichia coli* (*E. coli*) 1139#	This work
ECO-2	*E. coli* 2219#	This work
ECO-3	*E. coli* ZY-1	This work
ECO-4	*ATCC 700926*	(Brynildsen et al, 2013)

Appendix Table S2. Drug sensitivity of clinical isolated multidrug-resistant Gram-negative strains

Antibiotic	KP-1	KP-2	AB-1	AB-2	PA-1	PA-2	ECL-1	ECL-2	ECO-1	ECO-2	ECO-4
Amikacin	R	S	R	R	S	R	S	S	S	S	S
Ampicillin	R	R	R	R	R/S	R	/	/	S	R	S
Aztreonam	R	R	R	R	R	R	R	R	R	R	S
Cefazolin	R	R	R	R	R	R	/	/	R	R	S
Cefepime	R	R	R	R	R	R	R	R	R	R	S
Cefotaxime	R	R	R	R	R	R	R	R	R	R	S
Ceftazidime	R	R	R	R	R	R	R	R	R/S	R	S
Chloramphenicol	R	R	R	R	/	R	R	R	S	S	S
Ciprofloxacin	R	R	R	R	R	R	R	R	R	R	S
Gentamicin	R	R	R	R	S	R	R	R	S	R	S
Imipenem	R	S	R	R	S	/	S	R	S	S	S
Levofloxacin	R	R	R	R	R	R	R	R	R	R	/
Meropenem	R	S	R	R	R	R/S	S	R	S	S	S
Piperacillin	R	R	R	R	R/S	R	R	R	R	R	S
Polymyxin	/	/	S	S	S	S	R	S	/	/	/
Sulbactam	R	R	R	/	/	/	/	/	R/S	R	S
Sulfanilamide	S	R	/	S	R	R	R	R	R	R	S
Tazobactam	R	R	R	R	R/S	R	S	R	S	R/S	S
Tetracycline	S	R	R	R	R	R	R	R	R	S	S

*R: resistant; S: sensitive.

Appendix Table S3. ***Escherichia coli*** **DHB4 redox phenotypes used in the work**

**Strain**	**Genotype**	**Source**
Wild type	DHB4 (F′ *lac-pro lacI*^{Q}/ *Δ(ara-leu)7697 araD139 ΔlacX74 galE galK rpsL phoR Δ(phoA)*PvuII *ΔmalF3 thi*)	(Prinz et al, 1997)
trxA$^-$	DHB4 *ΔtrxA*	(Prinz et al, 1997)
trxB$^-$	DHB4 *trxB*::Kan	(Prinz et al, 1997)
trxC$^-$	DHB4 *ΔtrxC*	(Stewart et al, 1998)
trxA$^-$*trxB*$^-$*trxC*$^-$	DHB4 *ΔtrxA ΔtrxC trxB::Kan nadB::Tn*	(Stewart et al, 1998)
oxyR$^-$	DHB4 *oxyR*::Kan	(Vlamis-Gardikas et al, 2002)
gshA$^-$	DHB4 *gshA20*::Kan	(Prinz et al, 1997)
trxA$^-$*gshA*$^-$	DHB4 *ΔtrxA gshA20*::Kan	(Prinz et al, 1997)
gor$^-$	DHB4 *gor522*…mini-Tn10Tc	(Prinz et al, 1997)
gor$^-$*grxA*$^-$*B*$^-$*C*$^-$	DHB4 *gor522gxA*::Kan *grxB*::Kan mini-Tn10Tc *grxC*::Cm	(Aslund et al, 1999)
grxA$^-$*trxA*$^-$	DHB4 *ΔtrxA grxA*::Kan	(Prinz et al, 1997)

Appendix Table S4. Drug sensitivity of clinical isolated *E. coli* ZY-1

**Antibiotic**	**ZY-1**
Amikacin	S
Ampicillin	S
Aztreonam	S
Cefazolin	S
Cefepime	S
Cefotaxime	S
Ceftazidime	S
Chloramphenicol	S
Ciprofloxacin	S
Gentamicin	S
Imipenem	S
Levofloxacin	S
Meropenem	S
Piperacillin	S
Polymyxin	/
Sulbactam	S
Sulfanilamide	R
Tazobactam	S
Tetracycline	R

References

Aslund F, Zheng M, Beckwith J, Storz G (1999) Regulation of the OxyR transcription factor by hydrogen peroxide and the cellular thiol-disulfide status. *Proc Natl Acad Sci U S A* **96:** 6161-6165

Brynildsen MP, Winkler JA, Spina CS, MacDonald IC, Collins JJ (2013) Potentiating antibacterial activity by predictably enhancing endogenous microbial ROS production. *Nature biotechnology* **31:** 160-165

Prinz WA, Aslund F, Holmgren A, Beckwith J (1997) The role of the thioredoxin and glutaredoxin pathways in reducing protein disulfide bonds in the Escherichia coli cytoplasm. *The Journal of biological chemistry* **272:** 15661-15667

Stewart EJ, Aslund F, Beckwith J (1998) Disulfide bond formation in the Escherichia coli cytoplasm: an in vivo role reversal for the thioredoxins. *The EMBO journal* **17:** 5543-5550

Vlamis-Gardikas A, Potamitou A, Zarivach R, Hochman A, Holmgren A (2002) Characterization of Escherichia coli null mutants for glutaredoxin 2. *The Journal of biological chemistry* **277:** 10861-10868
