# Supplementary material for: Synergistic antibacterial effect of silver and ebselen against multidrug‐resistant Gram‐negative bacterial infections
Source: EMBO Mol Med. 2017 Jun 12;9(8):1165–78. doi: 10.15252/emmm.201707661 (PMC5538294; doi:10.15252/emmm.201707661)
Supplement: Supplementary file 2 — Expanded View Figures PDF [file EMMM-9-1165-s002.pdf]

Expanded View Figures

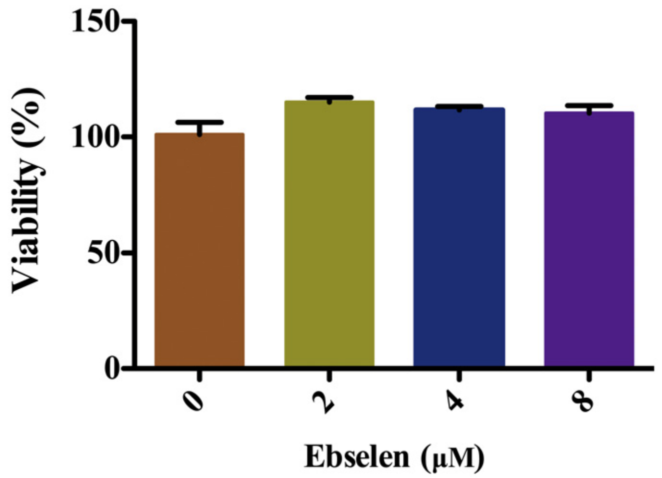

Figure EV1. Effects of ebselen on the growth of *E. coli*.
Escherichia coli DHB4 overnight cultures were diluted 1:1,000 into 100 μl of LB medium in 96 micro-well plates and treated with different concentrations of ebselen for 16 h. The cell viability was determined by measuring the absorbance at 600 nm. Data are presented as means ± SD of three independent experiments.

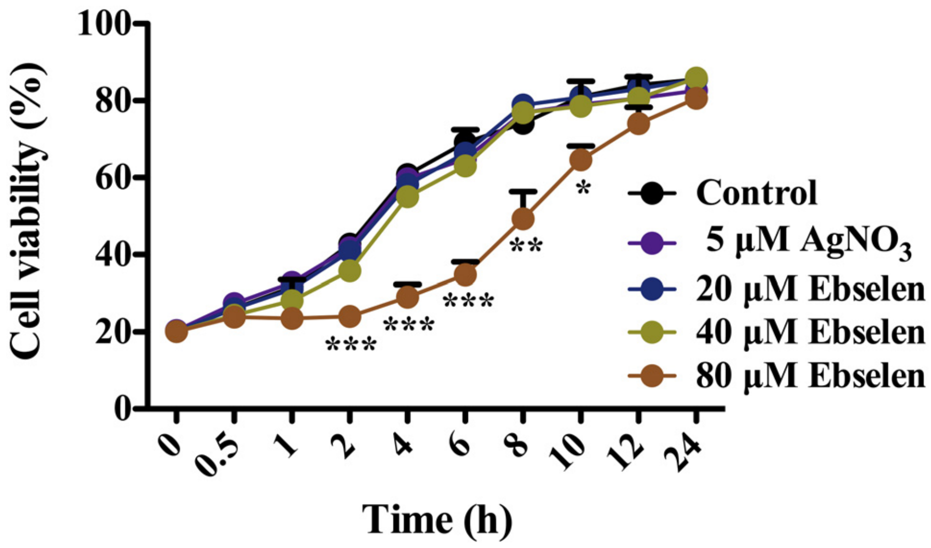

Figure EV2. Antibacterial effect of ebselen on *E. coli* growth.
Escherichia coli DHB4 cells were grown in 15-ml tubes until an $OD_{600\ nm}$ of 0.4 and treated with serial concentrations of ebselen for 24 h. The cell viability was determined by measuring the absorbance at 600 nm. Data are presented as means ± SD of three independent experiments. *$P < 0.05$, **$P < 0.01$, ***$P < 0.001$ (Student's *t*-test).

© 2017 The Authors

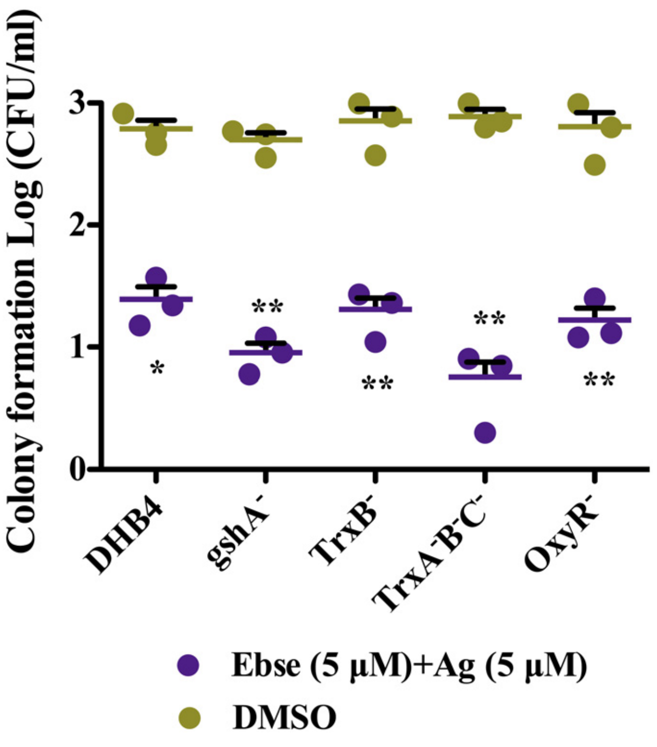

Figure EV3. Bactericidal effects of silver and ebselen in the LB medium containing heparinized mice blood.

Blood was extracted from three healthy mice and collected in heparinized tubes. One hundred *E. coli* DHB4 cells were harvested during the logarithmic phase, and drug combination was added to 100 μl heparinized mice blood. After incubation at 37°C for 6 h, duplicate 100-μl aliquots from each blood sample were spread onto LB agar, and CFU/ml was enumerated using the following formula: [(colonies) × (dilution factor)]/(amount plated) after overnight incubation. 0.1% (v/v) DMSO-treated cells were used as the positive control. Data are presented as means ± SD of three independent experiments. *$P < 0.05$, **$P < 0.01$ (Student's *t*-test).

 © 2017 The Authors

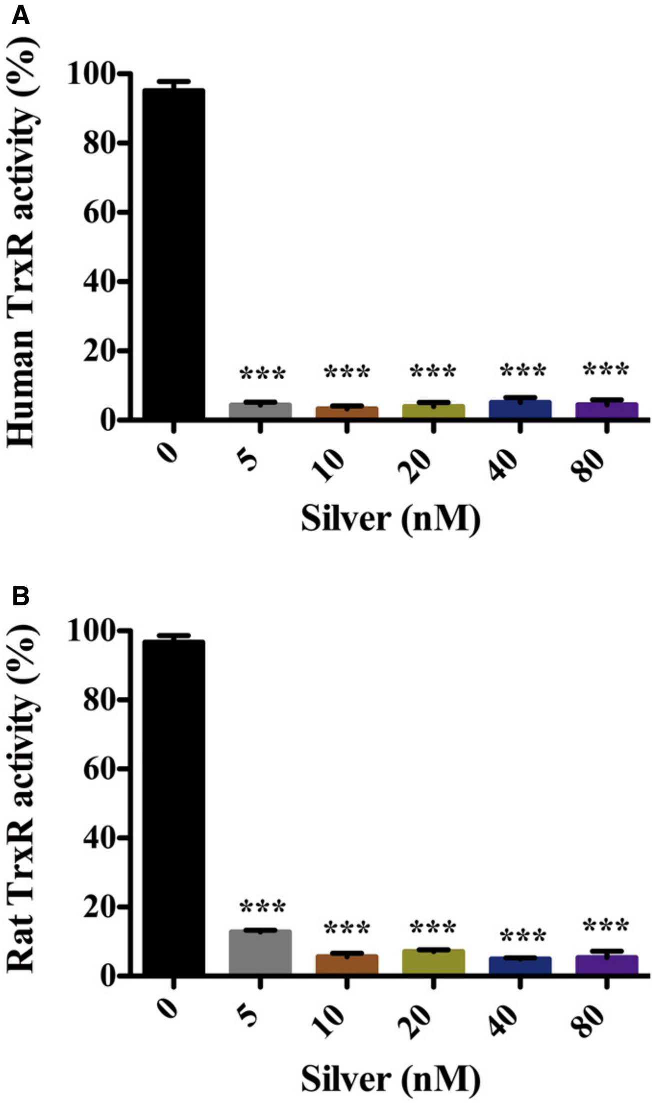

Figure EV4. Inhibitory effects of silver on mammalian TrxR *in vitro*.
Pure recombinant 10 nM human TrxR or 10 nM rat TrxR were incubated with serial concentrations of $AgNO_3$ solution in the presence of 250 μM NADPH, and then, their activities were detected by DTNB reduction assay.

A Inhibition of human TrxR by $AgNO_3$. 5 nM silver can inhibit human TrxR (***P = 0.000054).
B Inhibition of rat TrxR by $AgNO_3$. 5 nM silver can inhibit human TrxR (***P = 0.000019).

Data information: Data are presented as means ± SD of three independent experiments. ***$P < 0.001$ (Student's t-test).
